# Influence of metal ions on bioremediation activity of protocatechuate 3,4-dioxygenase from *Stenotrophomonas maltophilia* KB2

**DOI:** 10.1007/s11274-012-1178-z

**Published:** 2012-09-27

**Authors:** Urszula Guzik, Katarzyna Hupert-Kocurek, Karina Sałek, Danuta Wojcieszyńska

**Affiliations:** 1 Department of Biochemistry, Faculty of Biology and Environmental Protection, University of Silesia in Katowice, Jagiellonska 28, 40-032 Katowice, Poland; 2 Institute of Chemical Technology and Engineering, Poznan University of Technology, Pl. M. Skłodowskiej-Curie 2, 60-965 Poznań, Poland

**Keywords:** Aromatic compounds, Dioxygenases, Metal ions, *Stenotrophomonas*

## Abstract

The aim of this paper was to describe the effect of various metal ions on the activity of protocatechuate 3,4-dioxygenase from *Stenotrophomonas maltophilia* KB2. We also compared activity of different dioxygenases isolated from this strain, in the presence of metal ions, after induction by various aromatic compounds. *S. maltophilia* KB2 degraded 13 mM 3,4-dihydroxybenzoate, 10 mM benzoic acid and 12 mM phenol within 24 h of incubation. In the presence of dihydroxybenzoate and benzoate, the activity of protocatechuate 3,4-dioxygenase and catechol 1,2-dioxygenase was observed. Although Fe^3+^, Cu^2+^, Zn^2+^, Co^2+^, Al^3+^, Cd^2+^, Ni^2+^ and Mn^2+^ ions caused 20–80 % inhibition of protocatechuate 3,4-dioxygenase activity, the above-mentioned metal ions (with the exception of Ni^2+^) inhibited catechol 1,2-dioxygenase to a lesser extent or even activate the enzyme. Retaining activity of at least one of three dioxygenases from strain KB2 in the presence of metal ions makes it an ideal bacterium for bioremediation of contaminated areas.

## Introduction

Under aerobic conditions aromatic compounds are usually transformed to a few central intermediates such as catechols, protocatechuates, gentisates and (hydroxy)benzoquinols as a result of introduction of a new hydroxyl group at the *ortho*- or *para*-position to the existing one (Lillis et al. 2010; Guzik et al. 2011). Protocatechuic acid is a substrate for protocatechuate 3,4-dioxygenase [EC 1.13.11.3], enzyme catalyzing the intradiol cleavage of the aromatic ring, forming 3-carboxy-*cis*,*cis*-muconic acid (Contzen and Stolz 2000; Costas et al. 2004; Guzik et al. 2011). This enzyme belongs to a large class of the nonheme iron-containing dioxygenases and is composed of equimolar amounts of two α and β subunits (Ludwig et al. 1984; Buchan et al. 2000). The crystal structure of this enzyme shows that the high-spin iron(III) is bound to the active site in a distorted trigonal bipyramidal coordination geometry with two inequivalent tyrosine ligands, two histidines, and a hydroxide ion (Valley et al. 2005; Kurahashi et al. 2006; Mayilmurugan et al. 2010). The interaction of a substrate with Fe^3+^ causes the dissociation of both the axial tyrosine and the hydroxide resulting in a chelated “substrate-Fe^3+^” complex (Elgren et al. 1997; Vetting et al. 2000). This activates the substrate for an electrophilic attack by dioxygen, which leads to the formation of a peroxo bridge between iron and C4 of substrate (Pau et al. 2005; Borowski and Siegbahn 2006). Next, Criegee rearrangement (acyl migration to the peroxo oxygen) and O–O bond cleavage occurs, leading to the cyclic anhydride formation. The second atom of a molecular oxygen is retained at the Fe^3+^ as an oxide or hydroxide ion, where it functions as a nucleophile to hydrolyze the anhydride, yielding the ring open product (Vetting et al. 2000; Borowski and Siegbahn 2006). Due to the specific structure and mechanism of protocatechuate 3,4-dioxygenase-catalyzed reaction, the metal ions could influence the activity of this enzyme. During metalloenzymes catalysis, metal ions such as ferrum or calcium, are known to be the activators, since they induce conformational changes in the enzyme to stabilize the bound Fe^2+^ or to assist the orientation of catalysis site for a substrate binding (Ha et al. 2000). Gopal et al. (2005) suggested that the replacement of the iron in the active site with different metal ions caused the modulation of enzyme activity in accordance with the Irving-Williams order for bivalent metal ions. Several ions are known to be sulfhydryl groups` inhibitors and therefore change the conformation of a protein structure (Ha et al. 2000). In this paper we described the effect of various metal ions (Fe^2+^, Fe^3+^, Cu^2+^, Zn^2+^, Co^2+^, Al^3+^, Cd^2+^, Ni^2+^ and Mn^2+^) on the activity of protocatechuate 3,4-dioxygenase from *Stenotrophomonas maltophilia* KB2. As in strain KB2 induction of various types of dioxygenases was observed (Guzik et al. 2009), we compared the activity of these enzymes in the presence of different metal ions. Results of our studies seem to be very important for biodegradation processes since the metal ions present in the environment play an important role in bioremediation of aromatic compounds.

## Materials and methods

### Media and culture conditions for biodegradation of aromatic compounds

*Stenotrophomonas maltophilia* KB2 (VTT E-113197) is a gram-negative, aromatic compound-degrading bacterium isolated from the activated sludge of the sewage treatment plant in Bytom—Miechowice, Poland (Guzik et al. 2009; Wojcieszyńska et al. 2011b). 250 ml of the sterile MSM (Mineral Salt Medium) supplemented with 1 mM of the tested aromatic compound (phenol, protocatechuic acid, or benzoic acid) were inoculated with KB2 cells to the final optical density of about 0.1 in absorbance scale at λ = 600 nm (OD600), and incubated by shaking at 30 °C for 24 h. While growth of the cultures and complete degradation of the aromatic substrate was observed and OD600 of the culture was above 1.0, the proper volume of the culture was transferred to the new flask with sterile MSM to the final optical density of about 0.1 in absorbance scale at λ = 600 nm (OD600), the successive dose (2 mM and higher) of the aromatic substrate was added/introduced and the cultures were left for incubation for the next 24 h at 30 °C and 125 rpm. The residual aromatic compounds concentration in the culture filtrates was determined by the liquid chromatography.

Induction experiments were carried out in 1-l flasks, containing 500 ml of mineral salts medium and protocatechuic acid or benzoic acid at concentration of 6 and 10 mM, respectively. Protocatechuic acid and benzoic acid were used as the inducers of protocatechuate 3,4-dioxygenase and catechol 1,2-dioxygenase, respectively. Cells in the late exponential growth phase were used for enzymes isolation.

### Determination of aromatic compounds concentration

In order to study the degradation of the aromatic compounds, samples were taken periodically from the culture medium and centrifuged (6,000×*g*, 15 min). Concentration of aromatic compounds in the culture supernatant was determined by HPLC (Merck HITACHI) equipped with a LiChromospher^®^ RP-18 column (4 × 250 mm) and a DAD detector (Merck HITACHI). The wavelength for detection of substrates, composition of eluent and solvent as well as the flow rate were developed separately for each aromatic compound. The mobile phase, in phenol and benzoic acid determination was acetonitryl and water (50:50 v/v), in protocatechuic acid determination was methanol and 1 % acetic acid (25:75 v/v), at the flow rate of 1 ml·min^−1^. The detection wavelength was set at 285 nm for phenol and at 260 for benzoic and protocatechuic acids. Chemical compounds in the supernatant were identified and quantified by comparing HPLC retention times and UV-visible spectra with external standards.

### Preparation of crude enzymatic extract

Cells were harvested in the late exponential growth phase by centrifugation at 5,000×*g* for 15 min at 4 °C. The cells were then washed with 50 mM phosphate buffer, pH 7.0, and resuspended in the same buffer. The obtained cell extracts were sonicated 6 times for 15 s and centrifuged at 9,500×*g* for 20 min at 4 °C. The supernatant was used as a crude extract for enzyme assays.

### Enzyme assays

Activity of catechol 1,2-dioxygenase [EC 1.13.11.1] was measured spectrophotometrically by the formation of *cis*,*cis*-muconic acid at 260 nm (ε_260_ = 16,800 M^−1^ cm^−1^). The reaction mixture contained 20 μl of catechol (50 mM), 67 μl Na_2_EDTA (20 mM), 893 μl of phosphate buffer pH 7.4 (50 mM) and 20 μl of crude extracts in a total volume of 1 ml. Specific activity of protocatechuate 3,4-dioxygenase was assayed by measuring the consumption of oxygen. The reaction mixture contained 400 μl of protocatechuic acid (10 mM), 2,600 μl of phosphate buffer pH 7.2 (50 mM) and 1,000 μl of crude extract in a total volume of 4 ml according to Hou et al. (1976). Protein concentrations of the crude extracts were determined by the Bradford method (Bradford 1976).

### Effect of various metal ions on enzyme’s activity

The effects of metal ions on enzymes` activity were investigated using FeSO_4_, FeCl_3_, CuSO_4_, ZnCl_2_, CoCl_2_, AlCl_3_, CdSO_4_, NiCl_2_, and MnSO_4_. Protocatechuate 3,4-dioxygenase was preincubated in the phosphate buffer (50 mM, pH 7.2) containing: Fe^2+^, Fe^3+^, Cu^2+^, Zn^2+^, Co^2+^, Al^3+^, Cd^2+^, Ni^2+^, Mn^2+^ at a final concentration of 1–3 mM for 3 min at 30 °C. Effect of the metal ions on the activity of catechol 1,2-dioxygenase was studied by incubating it in the presence of above mentioned ions at concentration of 3 mM. After incubation, a residual enzymatic activity was measured as described above.

## Results and discussion

### Degradation of aromatic compounds by *S. maltophilia* KB2

**Fig. 1:**
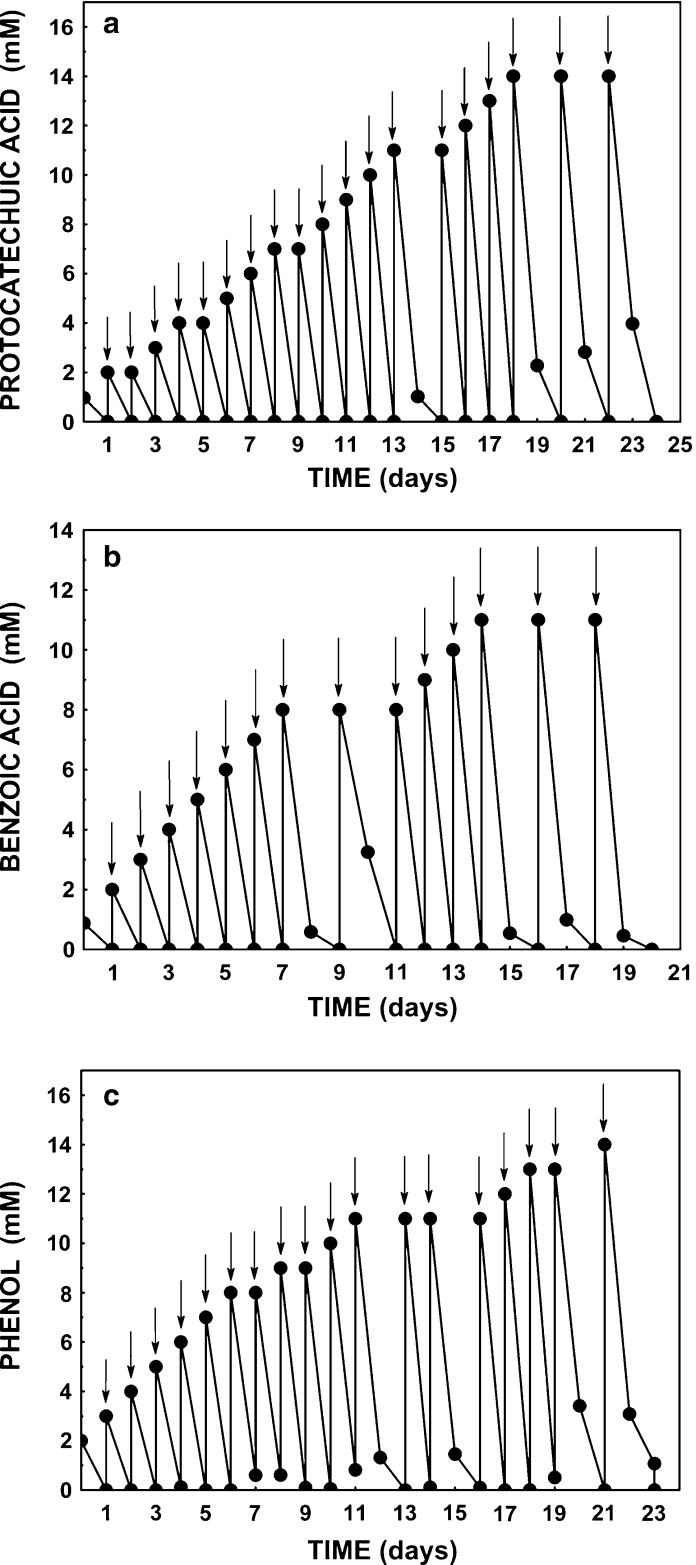
Adaptation of *Stenotrophomonas maltophilia* KB2 to utilize protocatechuic acid (**a**), benzoic acid (**b**), and phenol (**c**); *arrows* indicate introduction of growth substrate into the culture

*Stenotrophomonas maltophilia* KB2 is known to degrade a wide spectrum of aromatic compounds (Guzik et al. 2009; Greń et al. 2010; Wojcieszyńska et al. 2011b). In our previous works we found out that in this strain different dioxygenases were induced, depending on the aromatic substrate present in medium. In the presence of protocatechuic acid (3,4-DHB) strain KB2 synthesized protocatechuate 3,4-dioxygenase, while in the presence of benzoic acid (BA) and phenol (PH), activity of catechol 1,2-dioxygenase, and catechol 2,3-dioxygenase, respectively, was observed (Guzik et al. 2009; Wojcieszyńska et al. 2011a). It was interesting to examine the ability of KB2 strain to degrade even high concentrations of above-mentioned substrates: 13 mM of 3,4-DHB, 10 mM of BA, and 12 mM of phenol. As shown in Fig. 1a strain KB2 degraded up to 13 mM 3,4-DHB during 24 h. Significantly lower concentration of this substrate was degraded by *Moraxella* sp (2 mM) or *Burkholderia* sp. NCIMB 10467 (1,2 mM) (Sundman 1964; Sterjiades and Pelmont 1989). Additionally, no induction was required for the oxidation of protocatechuate by the latter strain (Sundman 1964; Luo et al. 2008).

Strain KB2 degraded 10 mM BA during 24 h (Fig. 1b) while *Streptomyces setonii* was capable of degrading only 5 mM of this substrate (An et al. 2000). Moreover, high concentrations of benzoate inhibited growth of *Pseudomonas putida* (Loh and Chua 2002). In contrast to the results obtained by Loh and Chua (2002), an inhibitory effect of benzoate was not observed by Muthukumar et al. (2009), during degradation of 25 mM benzoic acid by *Micrococcus* sp.

In our work we demonstrated that strain KB2 was also able to utilize 12 mM phenol during 24 h of incubation (Fig. 1c). Similar results were obtained by Shumakova et al. (2009) and Essam et al. (2010), who showed the ability of *Rhodococcus opacus* strain 1G and *Alcaligenes* strain TW1 to degrade 10 mM and 12 mM phenol, respectively. Degradation tests for the *Acinetobacter* strain ATTC11171 made by Adav et al. (2007) showed that this strain fully degraded 5 mM phenol, while at phenol concentrations higher than 5 mM, an inhibitory effect was observed. Phenol at low concentrations (1 and 2 mM) was degraded completely by *Ochrobactrum* AS1 and *Fusarium flocciferum* (El-Sayed et al. 2003; Mendonça et al. 2004). Our results show that strain KB2 exhibits ability to degrade a wide range of aromatic compounds at relatively high concentrations what makes it exceedingly attractive for industrial applications especially in bioremediation and wastewater treatment.

### Influence of metal ions on protocatechuate 3,4-dioxygenase activity

As it is generally known interactions between metal ions and residues in proteins are important for the protein’s stability, however metals are also known to be powerful inhibitors of the enzyme’s activity. Due to simultaneous contamination of industrial wastes by aromatic compounds and metals (Wu et al. 2008; Deeb and Altalhi 2009), there is an increasing interest in identification of enzymes that degrade aromatic structure and are resistant, among other factors, to the metal ions. In our study we examined influence of various metal ions on protocatechuate 3,4-dioxygenase, the non-heme iron—containing enzyme that catalyzes the *ortho* cleavage of the aromatic ring between the vicinal hydroxyls to form β-carboxy-*cis,cis*-muconic acid (Luo et al. 2008).

Addition of Zn^2+^, Co^2+^, Al^3+^, Cd^2+^ and Ni^2+^ ions caused a 20–80 % reduction of an initial protocatechuate 3,4-dioxygenase activity (Table 1). The presence of 1 and 2 mM of Mn^2+^ had no effect on the examined enzyme’s activity which could be explained by low toxicity of this ion (Nies 2000). About 70 % inhibition of the enzyme’s activity was observed after the addition of Cu^2+^ to the mixture reaction (Table 1). Inhibition of protocatechuate 3,4-dioxygenase caused by various metals was observed also by other authors (Nozaki et al. 1968; Hou et al. 1976; Iwagami et al. 2000). Metal ions can lead to the conformational changes in the enzymes such as a reduction in α-helices and β sheets, which can result in the loss of enzymatic activity (Latha et al. 2011). On the other hand, Gopal et al. (2005) observed no influence of Cu^2+^on the enzymatic activity of iron-containing quercetin 2,3-dioxygenase. They suggested that replacement of the iron at the active site of the enzyme with other metal may modulate its activity in accordance with the Irving-Williams studies on the stability of the metal complexes (Gopal et al. 2005; Matera et al. 2008). Obtained results allow us to assume that stability of the metal-enzyme complex formed in the active site of our enzyme was insufficient for its activity. Additionally, binding of transition metals ions such as Fe^3+^, Cu^2+^, Zn^2+^, Co^2+^, Cd^2+^, Ni^2+^, Mn^2+^ to the thiol groups of protocatechuate 3,4-dioxygenase might deactivate the enzyme. Moreover, protocatechuic acid, a substrate for protocatechuate 3,4-dioxygenase may form chelates with metals and these complexes are less accessible for the enzyme.

**Table 1:** Relative activity of protocatechuate 3,4-dioxygenase from strain KB2 in the presence of various metal ions (the plus/minus values represent standard deviation)

Compound	Concentration, mM	Relative activity, %
None	–	100 ± 0.00
Fe^2+^	1	43.00 ± 4.67
	2	119.93 ± 2.32
	3	128.92 ± 3.11
Fe^3+^	1	41.65 ± 5.72
	2	34.62 ± 1.49
	3	22.76 ± 2.11
Cu^2+^	1	41.81 ± 4.07
	2	27.36 ± 2.60
	3	29.64 ± 0.63
Zn^2+^	1	94.27 ± 7.21
	2	71.79 ± 5.17
	3	50.95 ± 2.76
Co^2+^	1	74.34 ± 19.14
	2	70.58 ± 8.45
	3	51.55 ± 4.69
Al^3+^	1	89.99 ± 5.43
	2	68.39 ± 3.21
	3	33.50 ± 2.14
Cd^2+^	1	69.33 ± 4.01
	2	63.85 ± 2.14
	3	60.20 ± 12.65
Ni^2+^	1	78.65 ± 3.02
	2	88.34 ± 2.29
	3	83.34 ± 0.31
Mn^2+^	1	108.06 ± 8.19
	2	98.55 ± 5.08
	3	80.92 ± 5.43

Intradiol dioxygenases contain a trivalent metal ion that is coordinated by four strictly conserved amino acid residues and one solvent molecule (Valley et al. 2005; Matera et al. 2008). A typical characteristic of most *ortho*-fission dioxygenases described so far is the increase of its activity by the addition of Fe^3+^ (Yeom and Yoo 1997; Iwagami et al. 2000). Iwagami et al. (2000) and Wu et al. (2008) stated that in metal ion catalysis, metals act as activators which induce conformational changes of the enzyme to stabilize it or support substrate binding by providing correct orientation of the catalytic site. However, we did not observe this effect. As shown in Table 1, the protocatechuate 3,4-dioxygenase from strain KB2 was inhibited by the addition of Fe^3+^. Similar effect was observed by Yeom and Yoo (1997) for *Alcaligenes xylosoxidans* catechol 1,2-dioxygenase. Transition metals such as Fe^3+^ rapidly react with sulfhydryl groups of cysteine residues which in turn affect tertiary structure of the enzyme. Partial loss of protocatechuate 3,4-dioxygenase’s activity in the presence of ferric ions might be caused by binding of Fe^3+^ ion to a site other than the active site of enzyme resulting in conformational changes in protein structure. To our surprise, the Fe^2+^ ions activated the examined enzyme. Similar effect was also observed by Yeom and Yoo (1997) in studies on the influence of metal ions on catechol 1,2-dioxygenases from *A. xylosoxidans* Y234. The influence of Fe^2+^ on protocatechuate 3,4-dioxygenase remains unsolved and needs further investigations.

### Effects of various metal ions on dioxygenases’ activity

*Stenotrophomonas maltophilia* KB2 synthesizes three types of dioxygenases depending on the aromatic substrate used (Guzik et al. 2009). Two of them (catechol 1,2-dioxygenase and protocatechuate 3,4-dioxygenase) belong to the intradiol dioxygenases family composed of enzymes with trivalent iron coordinated by one molecule of water, two histidine and two tyrosine residues, and their active sites are structurally very similar. In contrast catechol 2,3-dioxygenase from this strain is a member of extradiol dioxygenases family which comprises enzymes with bivalent iron in the active site (Vetting et al. 2000; Guzik et al. 2011; Wojcieszyńska et al. 2011b). The ability of strain KB2 to induce three types of dioxygenases makes it a very useful tool in bioremediation processes. Since contamination of the environment with aromatic compounds only is extremely rare it was very interesting to study the influence of metals at 3 mM concentration on the activity of dioxygenases isolated from this strain. Our previous studies on catechol 2,3-dioxygenase from strain KB2 shown activation of this enzyme in the presence of Zn^2+^ while Fe^2+^, Fe^3+^, Co^2+^, Cu^2+^, Al^3+^, Cd^2+^, Ni^2+^, Mn^2+^ reduced its activity (Guzik et al. 2010). Ni^2+^ and Cu^2+^ caused almost total loss of catechol 2,3-dioxygenase activity. Contrary to catechol 2,3-dioxygenase, almost all metal ions used in this study had no negative effects on catechol 1,2-dioxygenase from strain KB2 (Table 2). Similar results were obtained by Tsai and Li (2007) for Fe^2+^, Co^2+^, Cu^2+^ and Mn^2+^ions. Nevertheless, an inhibition of catechol 1,2-dioxygenase by metal ions was often observed (Hou et al. 1976; Yeom and Yoo 1997; Wang et al. 2006). Surprisingly, Cd^2+^ ions, which are known to be extremely toxic, caused high activation of catechol 1,2-dioxygenase (Table 2). The same effect was observed by Tokheim et al. (2005) for aryl sulfatase. Resistance of catechol 1,2-dioxygenase to Cd^2+^ seems to be very important, as these ions are frequent pollutants in aromatic hydrocarbons contaminated sites (Tsai et al. 2009; Stingu et al. 2012).

**Table 2:** Relative activity of dioxygenases from strain KB2 in the presence of various metal ions (the plus/minus values represent standard deviation)

Ion (3 mM)	Relative activity, %		
	Protocatechuate 3,4-dioxygenase	Catechol 1,2-dioxygenase	Catechol 2,3-dioxygenase (Guzik et al. 2010)
Control	100.00 ± 0.00	100.00 ± 0.00	100.00
Fe^2+^	128.92 ± 3.11	142.42 ± 3.21	72.29
Fe^3+^	22.76 ± 2.11	58.18 ± 8.33	67.15
Cu^2+^	29.64 ± 0.63	105.68 ± 17.68	0.50
Zn^2+^	50.95 ± 2.76	145.07 ± 2.68	118.00
Co^2+^	51.55 ± 4.69	162.12 ± 18.21	66.00
Al^3+^	33.50 ± 2.14	84.47 ± 4.82	72.00
Cd^2+^	60.20 ± 12.65	271.21 ± 26.78	75.00
Ni^2+^	83.34 ± 0.31	0.00 ± 0.00	14.00
Mn^2+^	80.92 ± 5.43	72.73 ± 2.14	46.00

The complete inhibition of catechol 1,2-dioxygenase activity was observed in the presence of Ni^2+^. As it is known this cation tends to bind to cysteine or histidine residues in the proteins (Nies 1999). Since histidines are the key residues forming the active site of the intradiol dioxygenases, the loss of catechol 1,2-dioxygenase activity was probably connected with interaction of Ni^2+^ with this amino acid. In spite of lower or even no activity of both: catechol 1,2- and 2,3-dioxygenase in the presence of nickel ions, strain KB2 could be still used in degradation of aromatic compound under these conditions due to the fact that protocatechuate 3,4-dioxygenase from this strain was minimally affected by Ni^2+^ (Table 2).

In summary, degradation of the selected aromatic compounds by *S. maltophilia* KB2 is catalyzed by one of three types of dioxygenases induced in this strain depending on the substrate used. These enzymes showed different sensitivity to the metal ions. Catechol 1,2- and 2,3-dioxygenase from this strain were strongly inhibited by Ni^2+^ ions while under the same conditions, protocatechutae 3,4-dioxygenase retained 80 % of its initial activity. In contrast Cu^2+^ ions inhibited protocatechuate 3,4-dioxygenase and catechol 2,3-dioxygenase while any negative effect of these ions on catechol 1,2-dioxygenase was observed. Induction of three types of dioxygenases in *S. maltophilia* KB2 ensure a degradation of aromatics which are present in the environment simultaneously contaminated with various metal ions.
